# Advanced Glycation End-Products Induce Apoptosis in Pancreatic Islet Endothelial Cells via NF-κB-Activated Cyclooxygenase-2/Prostaglandin E_2_ Up-Regulation

**DOI:** 10.1371/journal.pone.0124418

**Published:** 2015-04-21

**Authors:** Kuo-Cheng Lan, Chen-Yuan Chiu, Chia-Wei Kao, Kuo-How Huang, Ching-Chia Wang, Kuo-Tong Huang, Keh-Sung Tsai, Meei-Ling Sheu, Shing Hwa Liu

**Affiliations:** 1 Department of Emergency Medicine, Tri-Service General Hospital, National Defense Medical Center, Taipei, Taiwan; 2 Institute of Toxicology, College of Medicine, National Taiwan University, Taipei, Taiwan; 3 Department of Urology, College of Medicine and Hospital, National Taiwan University, Taipei, Taiwan; 4 Department of Pediatrics, College of Medicine and Hospital, National Taiwan University, Taipei, Taiwan; 5 Departments of Laboratory Medicine, College of Medicine and Hospital, National Taiwan University, Taipei, Taiwan; 6 Institute of Biomedical Sciences, National Chung Hsing University, Taichung, Taiwan; 7 Department of Medical Research, China Medical University Hospital, China Medical University, Taichung, Taiwan; National Centre for Scientific Research “Demokritos”, GREECE

## Abstract

Microvascular complications eventually affect nearly all patients with diabetes. Advanced glycation end-products (AGEs) resulting from hyperglycemia are a complex and heterogeneous group of compounds that accumulate in the plasma and tissues in diabetic patients. They are responsible for both endothelial dysfunction and diabetic vasculopathy. The aim of this study was to investigate the cytotoxicity of AGEs on pancreatic islet microvascular endothelial cells. The mechanism underlying the apoptotic effect of AGEs in pancreatic islet endothelial cell line MS1 was explored. The results showed that AGEs significantly decreased MS1 cell viability and induced MS1 cell apoptosis in a dose-dependent manner. AGEs dose-dependently increased the expressions of cleaved caspase-3, and cleaved poly (ADP-ribose) polymerase in MS1 cells. Treatment of MS1 cells with AGEs also resulted in increased nuclear factor (NF)-κB-p65 phosphorylation and cyclooxygenase (COX)-2 expression. However, AGEs did not affect the expressions of endoplasmic reticulum (ER) stress-related molecules in MS1 cells. Pretreatment with NS398 (a COX-2 inhibitor) to inhibit prostaglandin E_2_ (PGE_2_) production reversed the induction of cleaved caspase-3, cleaved PARP, and MS1 cell viability. Moreover, AGEs significantly increased the receptor for AGEs (RAGE) protein expression in MS1 cells, which could be reversed by RAGE neutralizing antibody. RAGE Neutralizing antibody could also reverse the induction of cleaved caspase-3 and cleaved PARP and decreased cell viability induced by AGEs. These results implicate the involvement of NF-κB-activated COX-2/PGE_2_ up-regulation in AGEs/RAGE-induced islet endothelial cell apoptosis and cytotoxicity. These findings may provide insight into the pathological processes within the pancreatic islet microvasculature induced by AGEs accumulation.

## Introduction

Diabetes mellitus (DM) is a multifactorial disease characterized by hyperglycemia and glucose intolerance due to insulin deficiency, impaired effectiveness of insulin action, or both [1]. Diabetic vascular complications are divided into two categories: macrovascular and microvascular complications. The atherosclerosis of large vessels is associated with macrovascular diseases in diabetes, which result in coronary artery diseases, stroke, and peripheral vascular diseases [2]. Microvascular complications include retinopathy, nephropathy, and neuropathy that eventually affect nearly all patients with diabetes [3]. Endothelial dysfunction is thought to play a prominent role in the pathogenesis of diabetic vascular complications. These complications are characterized by changes in proliferation, barrier function, adhesion of circulating cells, and sensitivity to apoptosis [4–6]. Moreover, evidence has demonstrated that endothelial cell apoptosis plays a crucial role in the development of early lesions in the microvasculature in patients with diabetes [1, 7]. Increased production of reactive oxygen species resulting in oxidative stress, cellular injury, and apoptosis occur in diabetes [8–10]. In addition to the role of reactive oxygen species, recent studies attempts to identify the role of inflammatory mediators in endothelial cell apoptosis during diabetes [1, 7]. In particular, cyclooxygenase-2 (COX-2) activation is associated with high glucose (hyperglycemia)-induced endothelial cell apoptosis and regulated by nuclear factor (NF)-κB signaling [11].

Hyperglycemia is the most important risk factor responsible for the development and progression of diabetic vascular complications [3, 12]. Advanced glycation end-products (AGEs) resulting from hyperglycemia are a complex and heterogeneous group of compounds that accumulate in the plasma and tissues in diabetic patients [13]. They are responsible for both endothelial dysfunction and diabetic vasculopathy [14–17]. The interaction between AGEs and receptor for AGEs (RAGE) elicits oxidative stress generation in various types of cells that leads to vascular inflammation, macrophage and platelet activation, and thrombosis, thereby the development and progression of vascular complications in diabetes [16, 18–20]. This AGE receptor ligation activates transcription factor NF-κB that leads to pathological changes in gene expression. This causes the production of inflammatory cytokines and growth factors, which in turn, cause vascular pathology [8].

In spite of a large number of DM-related studies focused mainly on organ-specific endothelial cells or human umbilical vein endothelial cells, the endothelium arising from vessels of different sizes and from different anatomical compartments expresses different phenotypic properties [21, 22]. Pancreatic islets are one of the most vascularized organs and are also influenced by diabetes, similar to the retina, kidney, and peripheral nervous system [23]. To address the potential pathogenic role of AGEs in microvascular complications of diabetes, we investigated the effects of AGEs on cytotoxicity and apoptosis induction in pancreatic islet microvascular endothelial cells. The results of our study provide an important insight for the role of inflammatory signaling induced by AGEs accumulation related to diabetic microvascular complications in pancreatic islets.

## Materials and Methods

### Reagents and antibodies

Anti-COX-2, anti-poly (ADP-ribose) polymerase (PARP), anti-GRP78, anti-GRP94, anti-RAGE, and secondary horseradish peroxidase-conjugated antibodies were obtained from Santa Cruz Biotechnology (Santa Cruz, CA, USA). Anti-phospho-p65, anti-caspase-3, anti-IRE1, anti-PERK, anti-ATF-6, anti-caspase-12, anti-phospho-p38, anti-Bcl-2, anti-Bax and anti-CHOP antibodies and RAGE neutralizing antibody were obtained from Cell Signaling Technology (Danvers, MA, USA). NS398 was obtained from Sigma-Aldrich Corporation (St. Louis, MO, USA).

### Preparation of AGEs

The preparation of AGEs was performed as described in our previous study [24]. In brief, BSA was incubated under sterile conditions with D-glucose in 0.2 M phosphate buffer (pH 7.4) at 37°C for 8 weeks. After incubation, AGEs were dialyzed against phosphate buffered saline for 24 h to remove unbound sugars and then filter-sterilized using a 0.22 μm Millipore filter (Millipore, Billerica, MA, USA). AGEs were identified using a MALDI-TOF/TOF mass spectrometry (Bruker, Billerica, MA, USA) and the AGE protein levels were measured using a bicinchoninic acid assay method.

### MS1 cell cultures

All experiments were performed using the mouse pancreatic islet endothelial cell line MS1 (Mile Sven 1 cells), which was purchased from the American Type Culture Collection. The MS1 cells were maintained and expanded on plastic tissue culture dishes in Dulbecco’s Modified Eagle Medium (DMEM, GIBCO, Life Technologies, Grand Island, NY, USA), pH 7.4, at 37°C in 5% CO_2_. The medium also contained 4 mM L-glutamine, 1.5 g/L sodium bicarbonate, 0.11 g/L sodium pyruvate, 4.5 g/L glucose, 5% fetal bovine serum, and standard tissue culture antibiotics. Cells were sub-cultured using trypsin-EDTA once the cells achieved 90% confluence about every 2 or 3 days.

### Cell viability assay

The yellow 3-(4,5-dimethylthiazol-2-yl)-2,5-diphenyltetrazolium bromide salt (MTT; Sigma-Aldrich) is reduced by mitochondrial succinate dehydrogenase in viable cells to form insoluble purple formazan crystals, which are soluble in dimethyl sulfoxide. Cells were seeded in 96-well culture plates. Cells were treated with AGEs for 24 h, and then stained with MTT (0.5 mg/mL) for 4 h. The media were removed and the produced formazan crystals were dissolved in 100 μL of dimethyl sulfoxide. The absorbance was measured at 570 nm.

### Annexin V-FITC apoptosis detection

MS1 cells were cultured in 6-cm dishes. Cells were treated with AGEs for 24 h, and then apoptosis was assessed using an annexin V-FITC apoptosis detection kit (Becton Dickinson, Franklin Lakes, NJ, USA). Cells were dissociated by 0.05% trypsin/EDTA for 3 min and then centrifuged at 1,000 rpm for 5 min and re-suspended in 100 μL of 1X binding buffer, and transferred into a 5-mL fluorescence-activated cell sorting (FACS) tube. Next, 5 μL of annexin V-FITC (conjugated with fluorescein isothiocyanate) and 5 μL of propidium iodide (PI) were added. After incubation for 30 min at room temperature in the dark, 400 μL of 1X binding buffer was added to each tube and the samples were immediately analyzed using a FACS flow cytometer.

### Western blot analysis

The total cell lysates were prepared. Equal amounts of proteins (25 μg per lane) were subjected to 10% sodium dodecyl sulfate-polyacrylamide gel electrophoresis followed by electrotransfer to polyvinylidene difluoride membranes (Millipore Corporation, Bedford, MA, USA). The membranes were blocked with 5% nonfat powdered milk in 0.1% Tris-buffered saline+Tween 20 for 1 h and probed with various primary antibodies at 4°C overnight. Subsequently, membranes were washed three times with 0.1% Tris-buffered saline+Tween 20 and incubated with secondary horseradish peroxidase-conjugated antibodies at room temperature for 1 h. After three washes, the signals were visualized using an enhanced chemiluminescence reagent detection system according to the manufacturer’s protocol (Millipore Corporation).

### PGE_2_ measurement

MS1 cells were cultured in 6-cm dishes. Cells were pre-incubated with 10 and 20 μM NS398 (COX-2 inhibitor) for 30 min, and then treated with 200 μg/mL AGEs for 24 h. The cell culture media were collected, and frozen at -80°C for measurement of PGE_2_ at a later time. PGE_2_ levels (pg/mL) were measured with a PGE_2_ enzyme immunoassay kit (Cayman Chemical, Ann Arbor, MI, USA), following the standard protocol enclosed with the kit. The PGE_2_ levels were normalized to the amount of cellular protein to which the conditioned media were exposed.

### Statistical analysis

Results are expressed as means ± SEMs for at least three independent experiments. The significant difference from the respective controls for each experimental test condition was assessed by one-way analysis of variance (ANOVA) and two-tailed Student's t-test. A value of *p* < 0.05 was considered statistically significant.

## Results

### Effects of AGEs on MS1 cell viability and apoptosis

**Fig 1:**
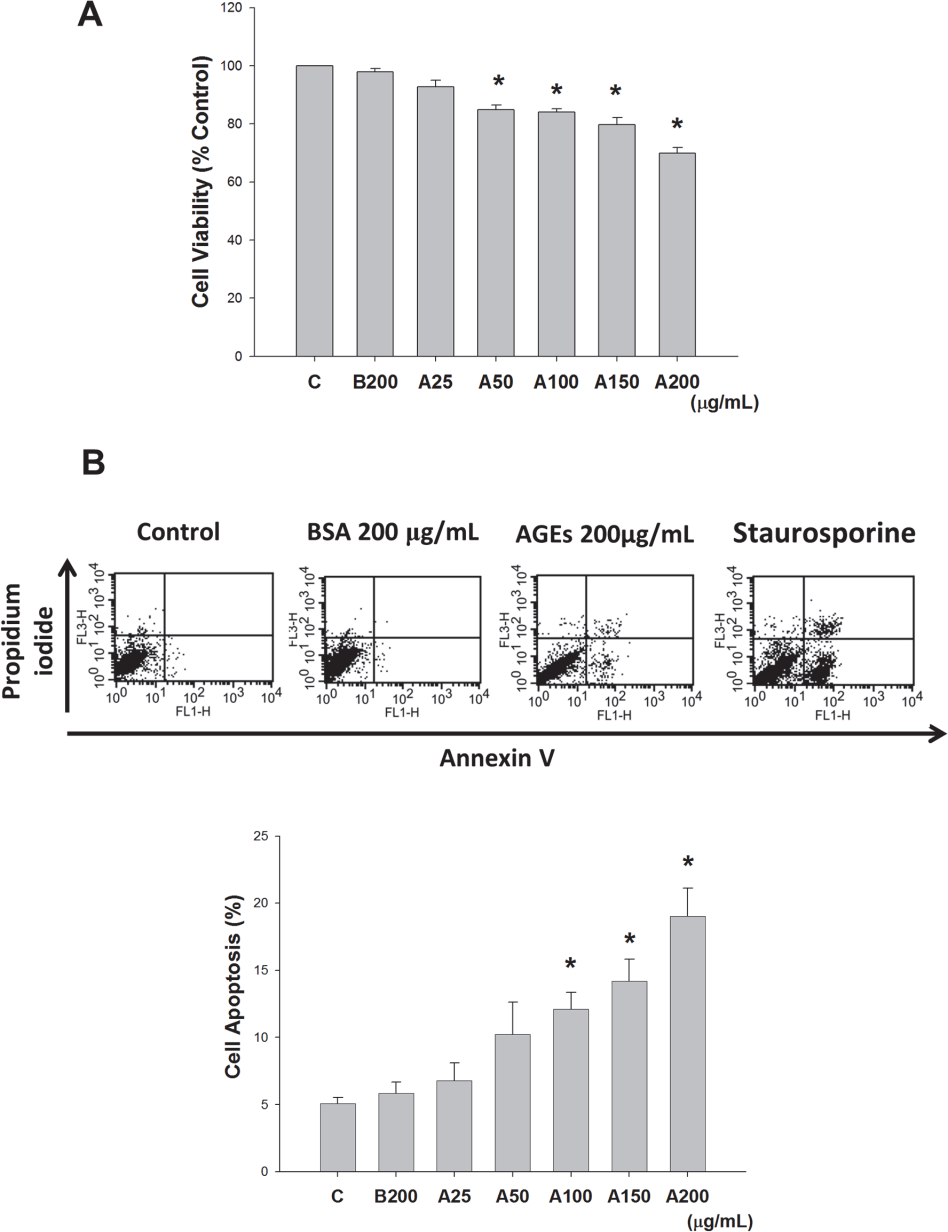
Advanced glycation end-products (AGEs) reduced cell viability and induced apoptosis. MS1 cells were treated with AGEs (A, 25, 50, 100, 150, and 200 μg/mL) and incubated for 24 h. Bovine serum albumin (BSA, B, 200 μg/mL) was as a negative control. The cytotoxicity was analyzed using the 3-(4,5-dimethylthiazol-2-yl)-2,5-diphenyltetrazolium bromide (MTT) assay (A). Cell apoptosis was measured using an annexin V-fluorescein isothiocyanate/propidium iodide binding assay (B). Staurosporine (200 μM) was as a positive control. Data are presented as means ± SEM from three independent experiments. *P < 0.05 vs BSA.

**Fig 2:**
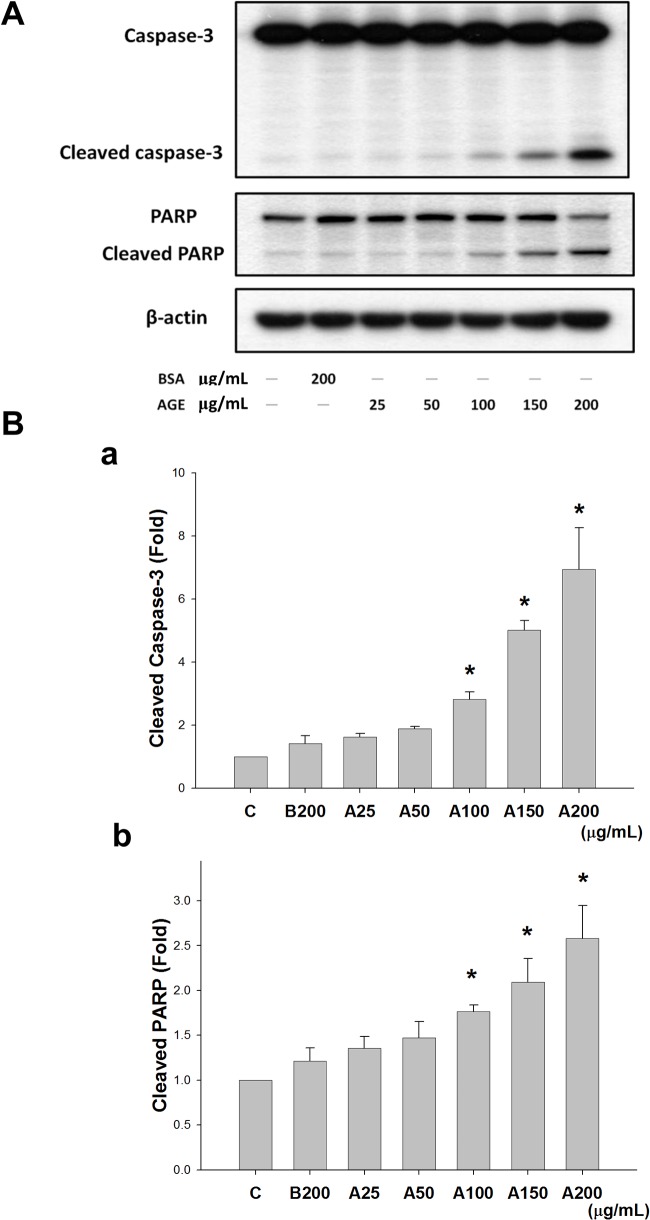
Advanced glycation end-products (AGEs) induced expression of cleaved caspase-3 and cleaved poly (ADP-ribose) polymerase (PARP) proteins. MS1 cells were treated with AGEs (A, 25, 50, 100, 150, and 200 μg/mL) for 24 h. Bovine serum albumin (BSA, B, 200 μg/mL) was as a negative control. Total cell lysates were then subjected to Western blot analysis using the indicated antibodies (A). β-actin was used as an internal standard. Densitometric analysis for protein expression was shown (B). Data are presented as means ± SEM from three independent experiments. *P < 0.05 vs BSA.

MS1 cells were treated with various concentrations of AGEs (25–200 μg/mL) for 24 h to examine the cytotoxic effect of AGEs. The viability of MS1 cells was significantly reduced in a dose-dependent manner (Fig 1A). Moreover, MS1 cells were incubated with various concentrations of AGEs (25–200 μg/mL) for 24 h to examine whether apoptosis was involved in AGEs-induced cytotoxicity. Exposure to AGEs statistically significantly increased the early and late apoptotic cell populations in a dose-dependent manner (Fig 1B). Similarly, protein expressions of cleaved caspase-3 and cleaved PARP in AGEs-treated MS1 cells were also increased in a dose-dependent manner (Fig 2). These results indicated that AGEs induced apoptosis in MS1 cells.

### Effects of AGEs on NF-κB-p65 phosphorylation and COX-2 protein expression

**Fig 3:**
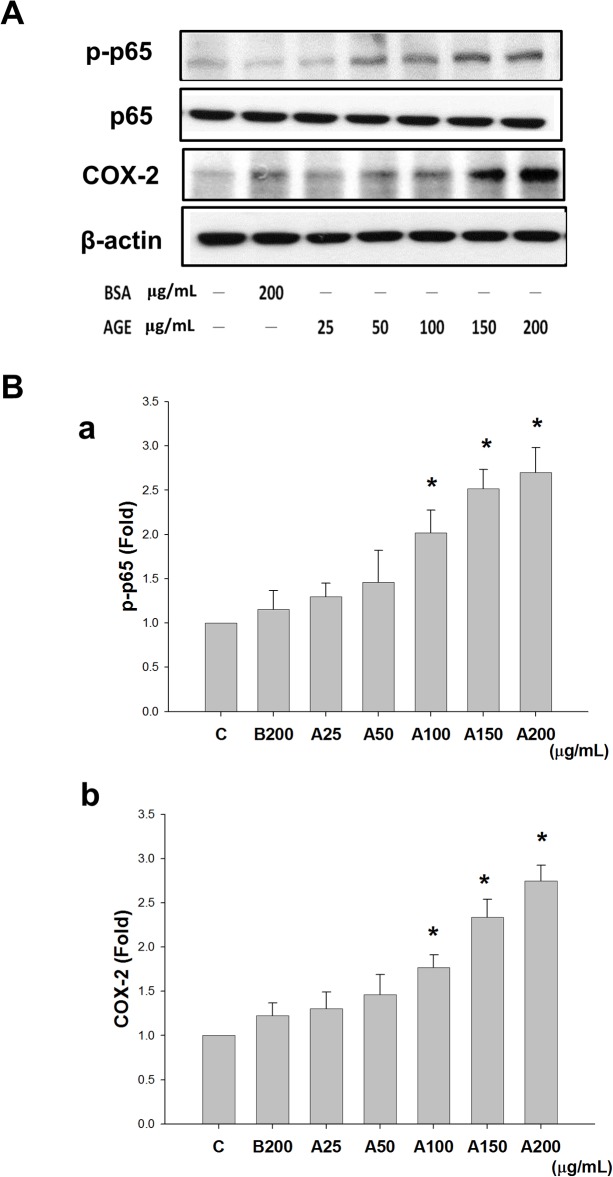
Advanced glycation end-products (AGEs) induced NF-κB-p65 phosphorylation and cyclooxygenase-2 (COX-2) protein expression. MS1 cells were treated with AGEs (A, 25, 50, 100, 150, and 200 μg/mL) for 24 h. Bovine serum albumin (BSA, B, 200 μg/mL) was as a negative control. Total cell lysates were then subjected to Western blot analysis using the indicated antibodies (A). β-actin was used as an internal standard. Densitometric analysis for protein expression was shown (B). Data are presented as means ± SEM from three independent experiments. *P < 0.05 vs BSA.

**Fig 4:**
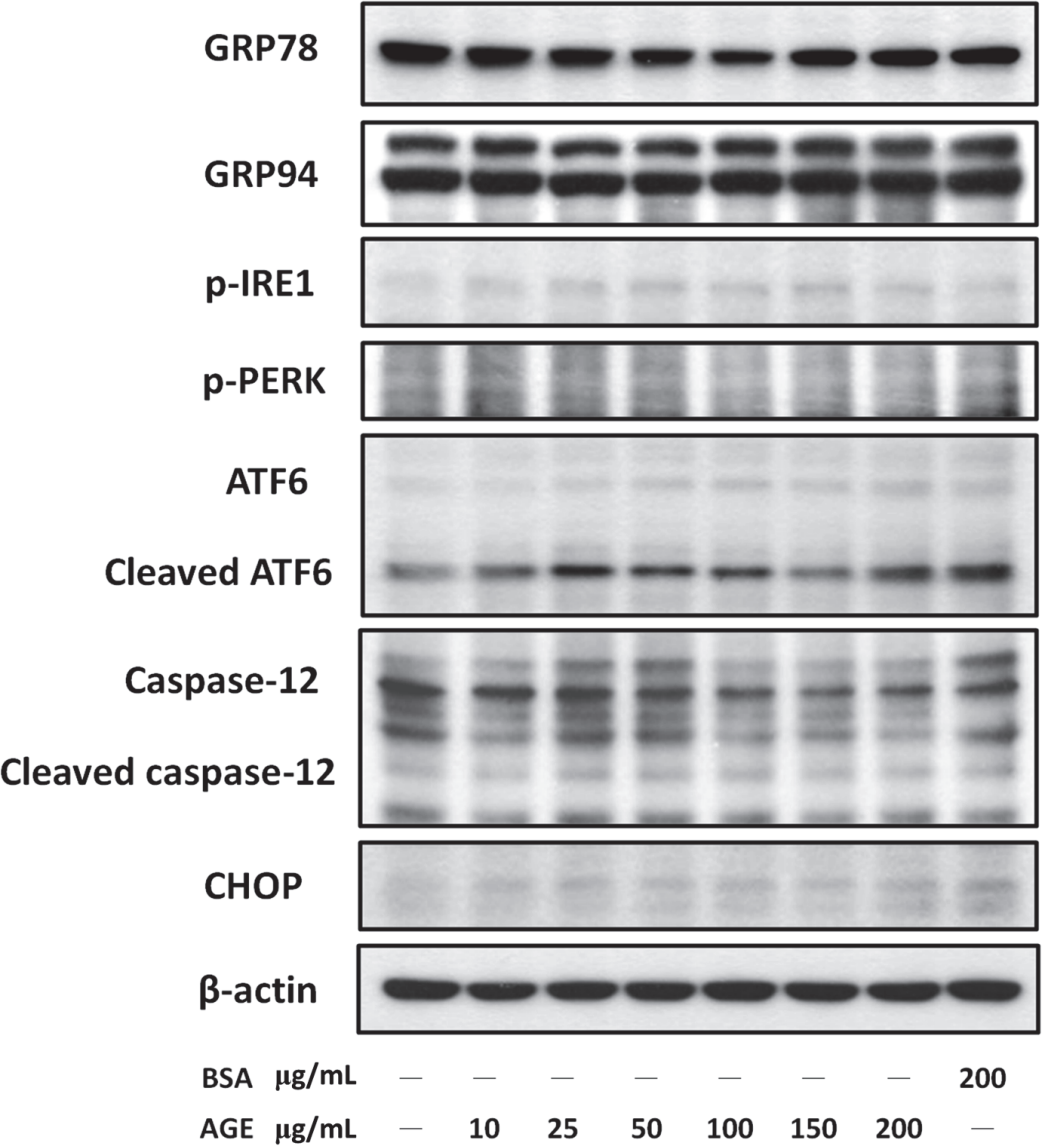
Effect of advanced glycation end-products (AGEs) on the expressions of endoplasmic reticulum (ER) stress-related molecules. MS1 cells were treated with AGEs (10–200 μg/mL) for 24 h. Bovine serum albumin (BSA, B, 200 μg/mL) was as a negative control. Total cell lysates were then subjected to Western blot analysis using the indicated antibodies. β-actin was used as an internal standard. Results shown are representative of three independent experiments.

MS1 cells were treated with AGEs (25–200 μg/mL) for 24 h to examine whether COX-2 played a role in AGE-induced apoptosis. The cell lysates were subjected to Western blotting for p65 phosphorylation and COX-2 protein expression. As shown in Fig 3, AGEs induced p65 phosphorylation and COX-2 protein expression in MS1 cells in a dose-dependent manner. The total p65 protein expression was not changed in AGEs-treated cells (Fig 3A). However, AGEs did not affect the protein expressions of endoplasmic reticulum (ER) stress-related molecules (GRP78, GRP94, IRE1, PERK, ATF-6, caspase-12, and CHOP) in MS1 cells (Fig 4).

### The role of COX-2/PGE_2_ in AGE-induced MS1 cell apoptosis

**Fig 5:**
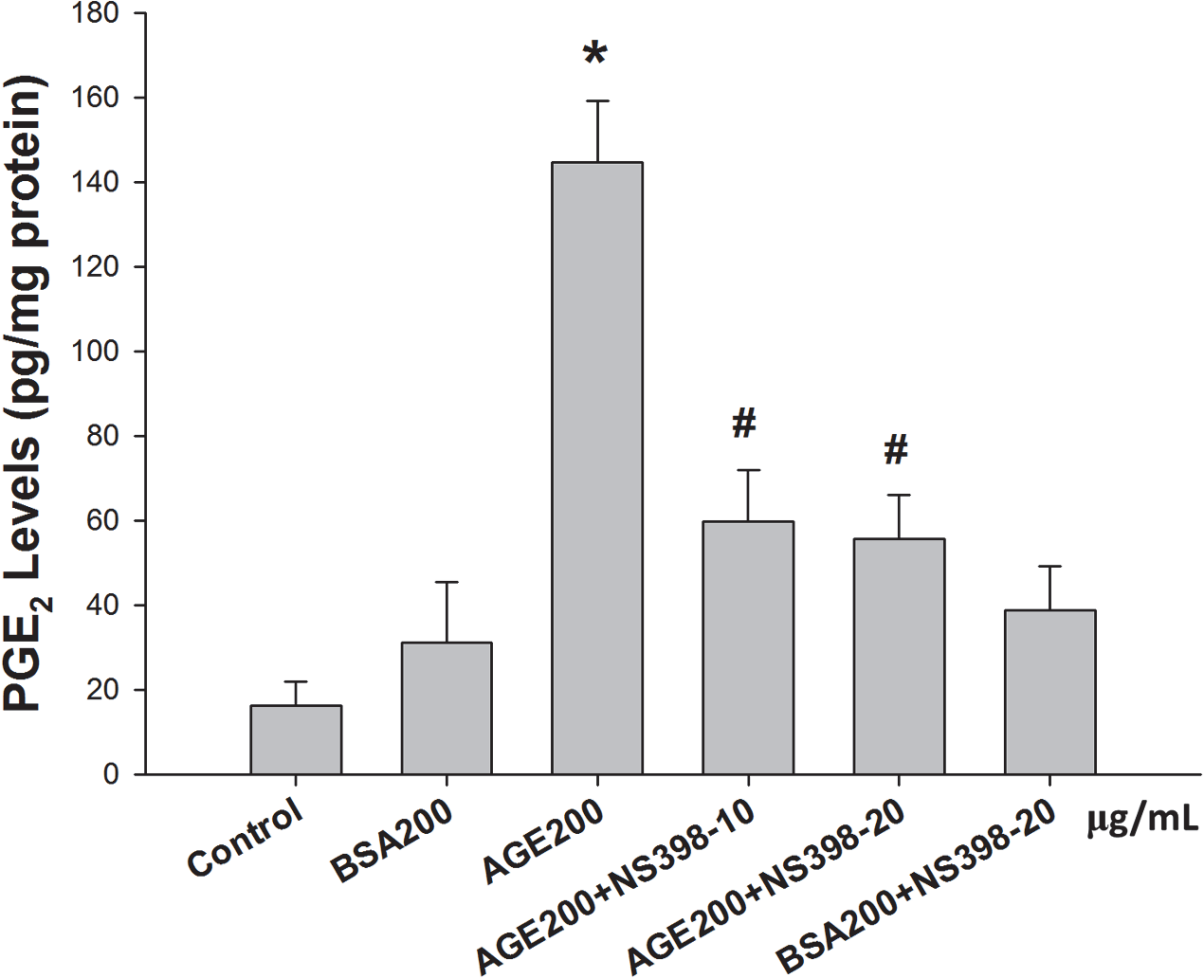
Advanced glycation end-products (AGEs) induced the production of prostaglandin-E_2_ (PGE_2_). MS1 cells were preincubated with NS398 (a cyclooxygenase-2 inhibitor, 10 and 20 μM) for 30 min, and then treated with AGEs (200 μg/mL) for 24 h. Bovine serum albumin (BSA, B, 200 μg/mL) was as a negative control. The cell culture medium subjected to PGE_2_ testing, and the PGE_2_ concentration was normalized to the amount of cellular protein to which the conditioned media was exposed. Data are presented as means ± SEM from three independent experiments. *P < 0.05 vs BSA; ^#^ *p* < 0.05 vs. AGEs.

**Fig 6:**
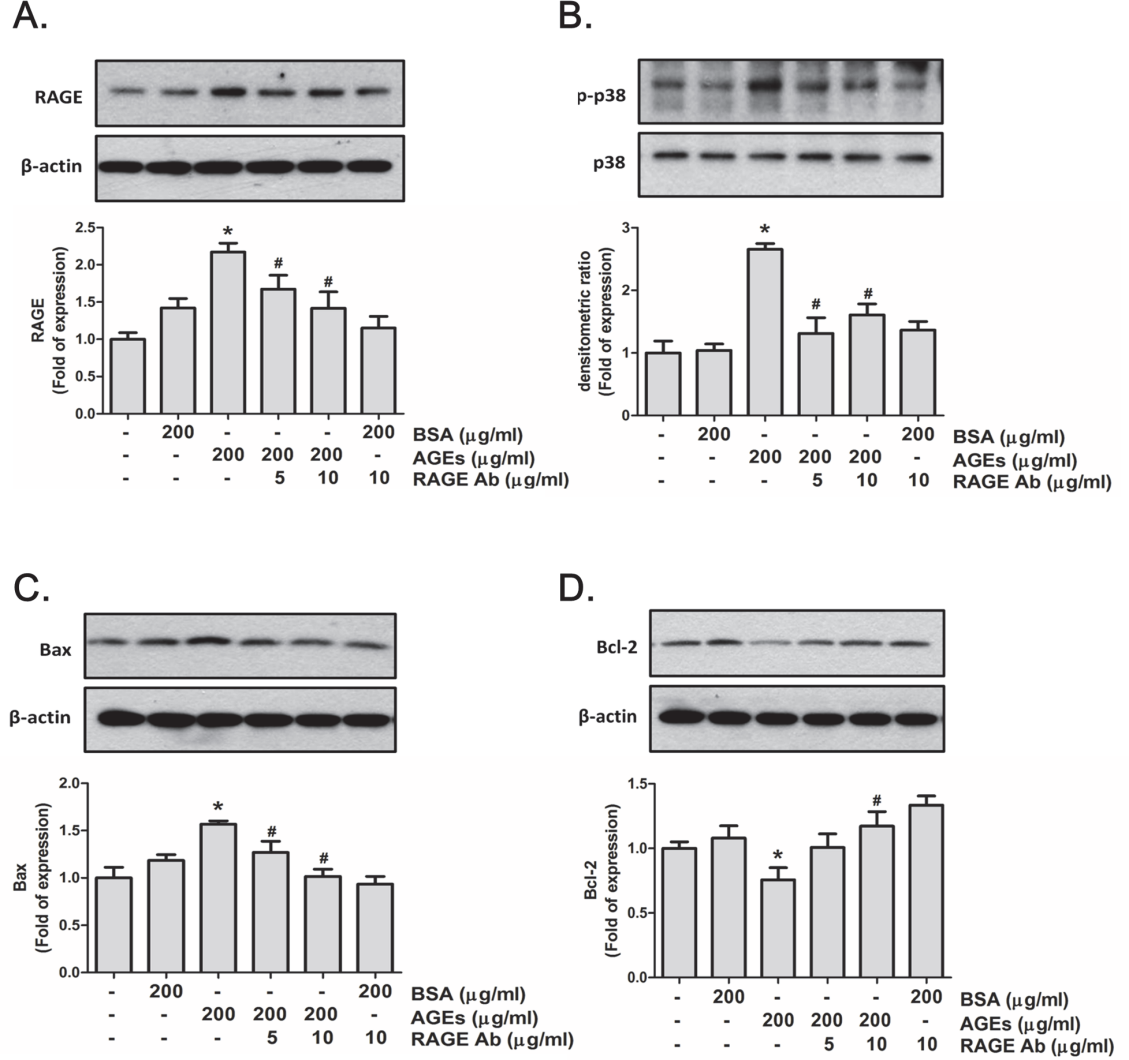
Advanced glycation end-products (AGEs) increased the expressions of RAGE, p38 MAPK, and Bax and decreased the Bcl-2 expression. MS1 cells were preincubated with RAGE neutralizing antibody (5 and 10 μg/mL) for 30 min, and then treated with AGEs (200 μg/mL) for 24 h. Bovine serum albumin (BSA 200 μg/mL) was as a negative control. Cell lysates were immunoblotted for RAGE (A), p38 MAPK (B), Bax (C), and Bcl-2 (D). β-actin served as an internal standard. Densitometric analysis for protein expression was shown. Data are presented as means ± SEM from three independent experiments. *P < 0.05 vs. bovine serum albumin (BSA) 200 μg/mL; #*p* < 0.05 vs. AGEs 200 μg/mL.

**Fig 7:**
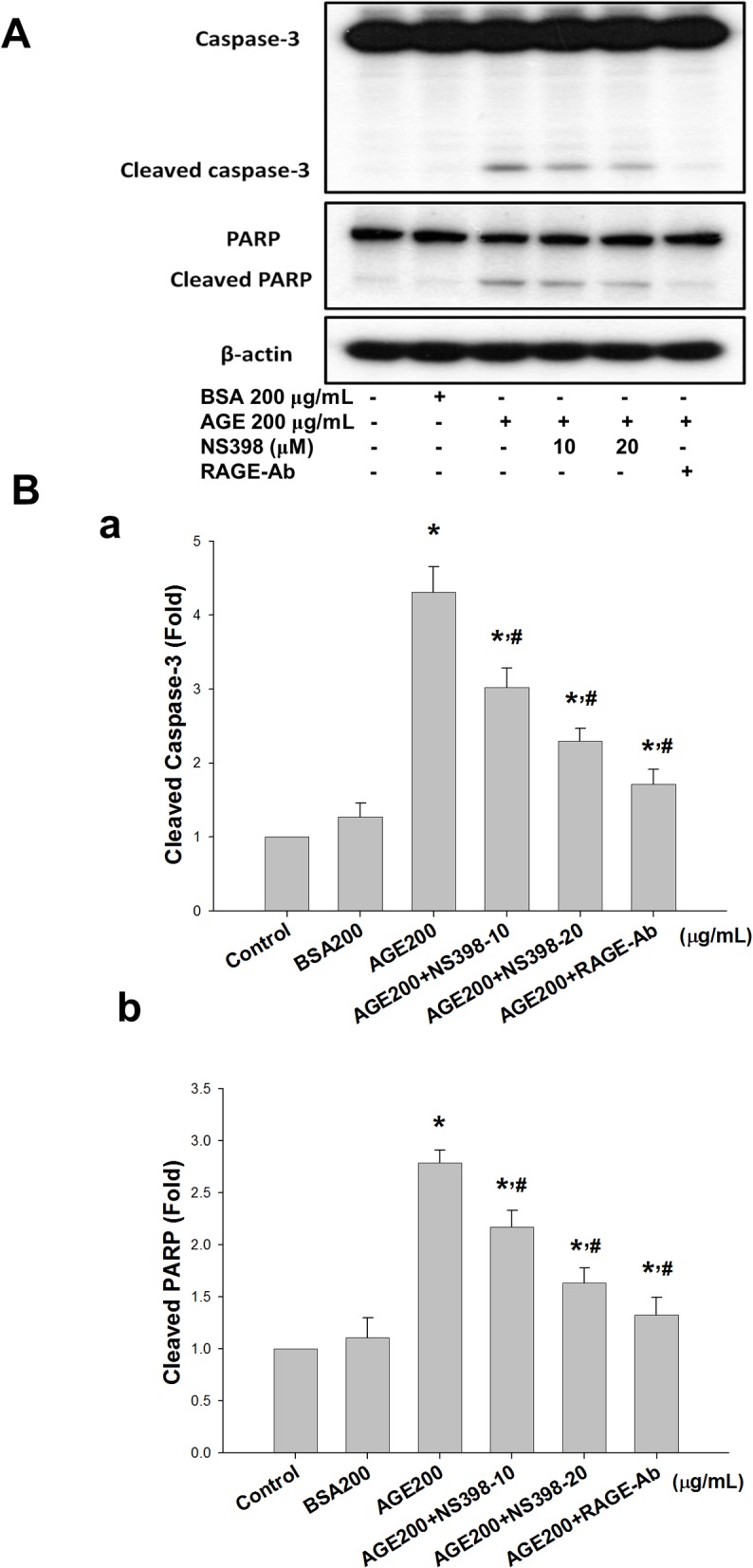
Cyclooxygenase-2 (COX-2) inhibitor and RAGE neutralizing antibody reversed advanced glycation end-products (AGEs)-induced protein expressions of cleaved caspase-3 and cleaved poly (ADP-ribose) polymerase (PARP). MS1 cells were preincubated with NS398 (10 and 20 μM) or RAGE neutralizing antibody (10 μg/mL) for 30 min, and then treated with AGEs (200 μg/mL) for 24 h. Bovine serum albumin (BSA, B, 200 μg/mL) was as a negative control. Cell lysates were immunoblotted for caspase-3 and PARP (A). β-actin served as an internal standard. Densitometric analysis for protein expression was shown (B). Data are presented as means ± SEM from three independent experiments. *P < 0.05 vs. bovine serum albumin (BSA) 200 μg/mL; ^#^ *p* < 0.05 vs. AGEs 200 μg/mL.

**Fig 8:**
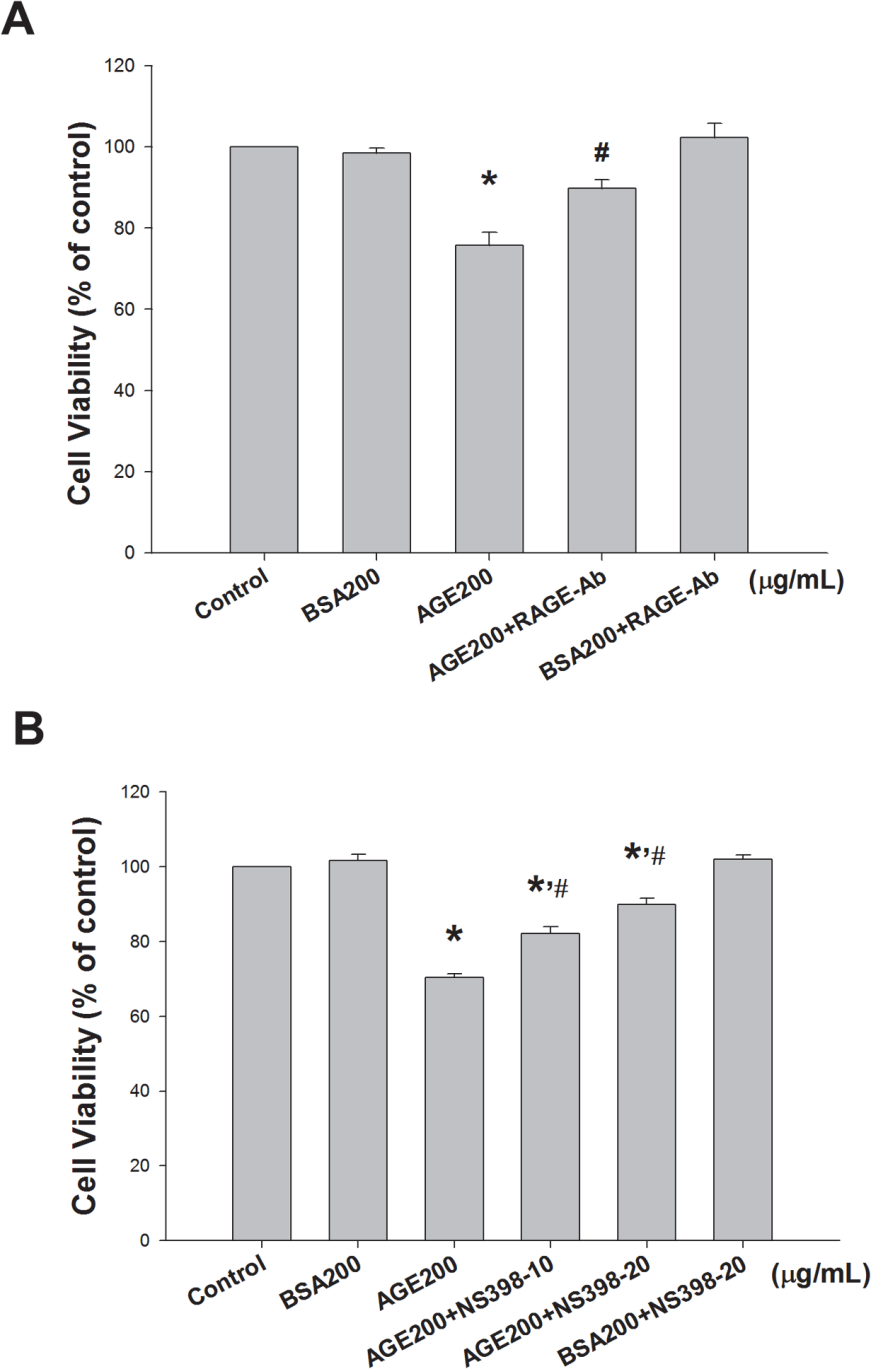
Cyclooxygenase-2 (COX-2) inhibitor and RAGE neutralizing antibody reversed advanced glycation end-products (AGEs)-reduced cell viability. MS1 cells were pre-incubated with RAGE neutralizing antibody (10 μg/mL, A) or NS398 (a COX-2 inhibitor; 10 and 20 μM, B) for 30 min, and treated with AGEs (200 μg/mL) for 24 h. Bovine serum albumin (BSA, B, 200 μg/mL) was as a negative control. The cell viability was analyzed using the 3-(4,5-dimethylthiazol-2-yl)-2,5-diphenyltetrazolium bromide (MTT) assay. Data are presented as means ± SEM from three independent experiments. *P < 0.05 vs BSA; ^#^ *p* < 0.05 vs AGEs.

NS398 (a COX-2 inhibitor) was used to confirm the role of COX-2 in AGE-induced MS1 cell apoptosis. MS1 cells were pretreated with 10 and 20 μM NS398 for 30 min followed by the administration of AGEs (200 μg/mL) for 24 h. The cell lysates and culture medium were subsequently subjected to Western blotting and PGE_2_ assay, respectively. The results showed that AGEs markedly increased the PGE_2_ production, which could be significantly reversed by NS398 in MS1 cells (Fig 5). On the other hand, we also investigated the role of RAGE in AGEs-related effects. We first tested the effect of AGEs on RAGE and mitochondria-dependent apoptosis-related protein expressions. As shown in Fig 6A, AGEs significantly increased the RAGE protein expression in MS1 cells, which could be reversed by RAGE neutralizing antibody. We next investigated the action of AGEs on mitochondria-dependent apoptosis signaling pathway and the effect of RAGE neutralizing antibody on this AGEs-induced response. AGEs significantly increased the phosphorylation of p38 MAPK and Bax protein expression and decreased the protein expression of Bcl-2, which could be reversed by RAGE neutralizing antibody (Fig 6B–6D). Moreover, both NS398 and RAGE neutralizing antibody significantly suppressed AGEs-induced expression of cleaved caspase-3 and cleaved PARP in MS1 cells (Fig 7). Pretreatment with RAGE neutralizing antibody (Fig 8A) or NS398 (Fig 8B) could also reverse the AGEs-reduced MS1 cell viability. These data suggest that AGEs/RAGE induced MS1 cytotoxicity and apoptosis, at least in part, via the increase in COX-2 activation and PGE_2_ production.

## Discussion

In this study, we demonstrated for the first time that AGEs could induce apoptosis, which led to decrease the cell viability, in pancreatic islet endothelial cells via a RAGE/NF-κB/COX-2/PGE_2_ signaling pathway. These findings have an important implication for the involvement of AGEs-induced microvascular endothelial cytotoxicity in diabetic pancreatic islets.

Accumulating evidence has demonstrated that AGEs cause cell apoptosis in various cells, including pancreatic β-cells, endothelial progenitor cells, and human umbilical vein endothelial cells [25–27]. AGEs can bind to RAGE on the endothelial cell membrane to trigger a signaling cascade (e.g. stimulating MAPK (p38, ERK1/2, or JNK) signaling, Rac/Cdc42) and lead to the up-regulation of the transcription factor NF-κB and its target genes [28]. It has also been shown that AGEs can induce caspase-3 activation and apoptosis via a MAPK-NF-κB signaling pathway in endothelial progenitor cells [26]. However, the studies about the apoptotic effect of AGEs/RAGE on pancreatic islet microvascular endothelial cells are relatively fewer. The results of present study also indicated that NF-κB appears to play a predominant role as a transcription factor for the effects of AGEs/RAGE on apoptosis in pancreatic islet microvascular endothelial cells. On the other hand, NF-κB activation is known to induce COX-2 up-regulation and contributes to inflammatory responses. While COX-2 is expressed at low levels in tissues and cells, it is significantly induced by inflammatory stimuli such as lipopolysaccharide, cytokines, and chemicals [29]. In the pancreatic islets, the induction of COX-2 leading to PGE_2_ production is believed to play a critical role in inflammation, islet destruction, and inhibition of insulin secretion [30, 31]. A previous study showed that a specific inflammatory ligand of RAGE, S100b, induced COX-2 expression and activity in both isolated human pancreatic islets and diabetic mouse models [32]. COX-2, which plays a crucial role in mediating inflammatory responses, is regulated by the NF-κB signaling pathway and promotes the synthesis of prostaglandins. NF-κB-activated COX-2/PGE_2_ up-regulation induced by β-amyloid has been shown to be associated with inflammatory cell death/apoptosis [11]. Moreover, RAGE is known to engage distinct classes of ligands, including AGEs and β-amyloid [33]. In the present study, the results showed AGEs-induced COX-2 protein expression and PGE2 production in pancreatic islet endothelial cells. COX-2 inhibitor NS398 inhibited the increases in COX-2 expression, PGE2 production, and caspase-3 expression, and PARP cleavage, and the decrease in cell viability by AGEs treatment. We also found that RAGE neutralizing antibody significantly suppressed AGEs-induced apoptosis and cytotoxicity. These findings suggest that NF-κB-activated COX-2/PGE_2_ up-regulation is involved in the AGEs/RAGE-induced islet endothelial cell apoptosis.

In the present study, we found that AGEs caused the activation of NF-κB/COX-2/PGE_2_ and induction of caspase-3, leading to cell apoptosis in islet microvascular endothelial cells; however, AGEs did not affect the protein expressions of ER stress-related molecules. The response of ER stress is known to constitute a cellular process, which can be induced by a variety of conditions, disturbing the folding of proteins in the ER. Accumulating evidence has indicated that ER stress signaling is involved in the cellular dysfunction and cell death, which are major contributors to many diseases [34]. ER stress-induced cell death has been shown to play a role in the pathogenesis of atherosclerosis and diabetic macrovascular complications [35]. A recent study has found that AGEs (AGE-BSA, 100 and 200 μg/mL) induce ER stress-related molecules, which are correlated with elevated apoptosis signal at the same time-points, in human aortic endothelial cells [36]. The results of present study indicated that AGE-BSA (200 μg/mL) did not trigger the ER stress response, but significantly induced apoptosis in pancreatic islet microvascular endothelial cell line MS1 cells. Taken together, these results implicated that ER stress signaling may not be involved in the AGEs-induced apoptosis in islet endothelial cells under the present experimental conditions.

Insulin resistance and islet β-cell dysfunction are important in the pathogenesis of glucose intolerance leading to type 2 diabetes [37]. Evidence indicates that AGEs are important mediators of β-cell dysfunction. Exposure of pancreatic β-cell lines and primary cultured islets from rats to AGEs resulted in increased apoptosis of the islet β-cells [25]. The pancreatic islet endothelium has unique structural and functional features and has an interdependent physical and functional relationship with neighboring β-cells [38]. Thus, we speculated that pancreatic endothelial cell dysfunction also contributed to the pathogenesis of the islets in type 2 diabetes. Our results showed that AGEs was capable of inducing apoptosis in islet microvascular endothelial cells. Therefore, the effect of AGEs on pancreatic islet endothelial cells not only affects the survival of β-cells within the islets, but also contributes to islet destruction resulting in the pathogenesis of diabetes mellitus.

It has been widely accepted that mitochondria is a crucial target in apoptotic process [39]. Recent evidence suggests that oxidative stress may trigger mitochondrial apoptotic cell death associated with activation MAPK pathway and mitochondrial pro-apoptotic molecule Bax expression [40]. Extensive evidence supports the fact that AGEs trigger cell apoptosis through the similar mitochondrial cascade signaling pathway. Shi et al. (2013) have reported that AGEs induce human corneal epithelial cell apoptosis via the activation of p38 MAPK/Bax/Bcl-2 signaling pathway [41]. Figarola et al. (2014) have also suggested that methylglyoxal, a reactive dicarbonyl precursor of AGEs, induced apoptosis in human umbilical vascular endothelial cells by activation of MAPK signaling and induction of Bax/Bcl-2 ratio [42]. In the present study, we found that AGEs induced a significant increase of phosphorylated p38 MAPK and Bax protein expressions and a marked inhibition of anti-apoptotic molecule Bcl-2 protein expression in MS1 cells, indicating that a mitochondria-dependent apoptotic pathway may also be involved in the AGEs-induced MS1 cell apoptosis.

In conclusion, this study provided evidence to determine the cellular pathological role of AGEs in pancreatic islet endothelial cells. AGEs that bind to its receptor (RAGE) lead to induce cell apoptosis via a NF-κB-activated COX-2/PGE_2_ up-regulation pathway. The results of this *in vitro* study provide insight into the pathological processes taking place within the pancreatic islet endothelium and AGEs-induced cytotoxicity in islet microvascular endothelial cells. Further investigations to understand the comprehensive effects of AGEs on the pancreatic islet endothelium are needed.
